# Semi-Solid Dosage Forms Containing Pranoprofen-Loaded NLC as Topical Therapy for Local Inflammation: In Vitro, Ex Vivo and In Vivo Evaluation

**DOI:** 10.3390/gels9060448

**Published:** 2023-05-29

**Authors:** Negar Ahmadi, María Rincón, Marcelle Silva-Abreu, Lilian Sosa, Jessica Pesantez-Narvaez, Ana Cristina Calpena, María J. Rodríguez-Lagunas, Mireia Mallandrich

**Affiliations:** 1 Departament de Farmàcia, Tecnologia Farmacèutica, i Fisicoquímica, Facultat de Farmàcia i Ciències de l’Alimentació, Universitat de Barcelona (UB), 08028 Barcelona, Spain; nahmadah7@alumnes.ub.edu (N.A.); silvadeabreu@ub.edu (M.S.-A.); anacalpena@ub.edu (A.C.C.); 2 Departament de Ciència de Materials i Química Física, Facultat de Química, Universitat de Barcelona (UB), 08028 Barcelona, Spain; 3 Institut de Nanociència i Nanotecnologia IN2UB, University of Barcelona, 08028 Barcelona, Spain; 4 Pharmaceutical Research Group, Faculty of Chemical Sciences and Pharmacy, National Autonomous University of Honduras (UNAH), Tegucigalpa 11101, Honduras; liliansosa2012@gmail.com; 5 Artificial Intelligence Applications (AIS), 08021 Barcelona, Spain; jessica-pesantez@live.com; 6 Department of Biochemistry and Physiology, Faculty of Pharmacy and Food Sciences, University of Barcelona, 08028 Barcelona, Spain; mjrodriguez@ub.edu; 7 Nutrition and Food Safety Research Institute (INSA-UB), 08921 Santa Coloma de Gramenet, Spain

**Keywords:** Carbomer 940, Sepigel^®^ 305, pranoprofen, nanostructured lipid carriers, drug delivery, inflammation, biomechanical properties

## Abstract

Pranoprofen (PRA)-loaded nanostructured lipid carriers (NLC) have been dispersed into blank gels composed of 1% of Carbomer 940 (PRA-NLC-Car) and 3% of Sepigel^®^ 305 (PRA-NLC-Sep) as a novel strategy to refine the biopharmaceutical profile of PRA, for dermal administration in the treatment of skin inflammation that may be caused by possible skin abrasion. This stratagem intends to improve the joining of PRA with the skin, improving its retention and anti-inflammatory effect. Gels were evaluated for various parameters such as pH, morphology, rheology, and swelling. In vitro drug release research and ex vivo permeation through the skin were carried out on Franz diffusion cells. Additionally, in vivo assays were carried out to evaluate the anti-inflammatory effect, and tolerance studies were performed in humans by evaluating the biomechanical properties. Results showed a rheological profile common of semi-solid pharmaceutical forms for dermal application, with sustained release up to 24 h. In vivo studies using PRA-NLC-Car and PRA-NLC-Sep in *Mus musculus* mice and hairless rats histologically demonstrated their efficacy in an inflammatory animal model study. No signs of skin irritation or modifications of the skin’s biophysical properties were identified and the gels were well tolerated. The results obtained from this investigation concluded that the developed semi-solid formulations represent a fitting drug delivery carrier for PRA’s transdermal delivery, enhancing its dermal retention and suggesting that they can be utilized as an interesting and effective topical treatment for local skin inflammation caused by a possible abrasion.

## 1. Introduction

The concept of abrasion means wearing down surfaces by friction. It is synonymous with erosion, wear, or chafing. In medicine, the term refers to the superficial ex-ulceration of the skin or mucous membranes by rubbing or scraping; that is, the procedure of mechanically removing different integument layers for cosmetic and/or therapeutic purposes [1,2,3,4,5]. An example of an abrasion application is depilation by chafing, a very useful hair removal method for people with certain skin sensitivities that make it difficult to remove hair using epilation systems such as waxes or special machines. For these cases, using systems such as silicon abrasion is especially interesting because it is easy to use and inexpensive. It is rubbed gently into dry skin until the hair is removed from the abrasion. With continued use of the system, the minerals gradually weaken the hair, just like when clothing is constantly rubbed against certain body parts, such as pants or stockings, and removes hair on the knees or thighs, or the typical bald spots people get from socks. Products such as silicone gloves make this easy and sensible hair removal possible. This product is made from a highly effective mineral that gently wears down body hair and deeply exfoliates the skin. Friction progressively undoes the hair; however, excessive or forceful abrasion can lead to local inflammation of the skin. 

The inflammatory process is a nonspecific immune response against external changes or cellular injury by harmful agents. This essential defense mechanism attempts to restore homeostasis to protect the injured tissues. Acute inflammation is the main and first response to exogenous factors caused by the collection of leukocytes that migrate from the blood to the injured areas [6,7]. When the body encounters something harmful (such as a virus, bacteria, or toxic chemical) or is injured, it activates the immune system. The immune system sends out its first responses: inflammatory cells and cytokines (substances that stimulate more inflammatory cells). These cells begin an inflammatory response to trap bacteria and other invading pathogens or to heal injured tissue. The result may be pain, swelling, bruising, or redness. Controlling the inflammatory response is critical to preventing irreversible tissue damage and controlling symptoms. Nonsteroidal anti-inflammatory drugs (NSAIDs) and corticosteroids are currently the most effective drugs for controlling the clinical manifestations of inflammation, although they often need to be combined with other “disease-modifying” strategies to achieve optimal control of chronic inflammatory diseases. As with any drug therapy, the benefit/risk balance of each drug must be carefully considered before initiating a longer course of treatment. Strategies to minimize the long-term risks of NSAIDs and corticosteroids are evolving and promise to improve safety without a loss of efficacy.

The primary mechanism of action of NSAIDs is the inhibition of cyclooxygenase (COX). Cyclooxygenase is required to convert arachidonic acid to thromboxane, prostaglandins, and prostacyclins [8]. The therapeutic effect of NSAIDs has been attributed to the deficiency of these eicosanoids. In particular, thromboxanes play a role in platelet adhesion, and prostaglandins cause vasodilation, increase the temperature set point in the hypothalamus, and play a role in antinociception. COX-1 is constitutively expressed in vivo and plays a role in maintaining gastrointestinal mucosa, renal function, and platelet aggregation. COX-2 is not constitutively expressed in vivo; instead, it is induced during inflammatory responses. Most NSAIDs are nonselective, inhibiting both COX-1 and COX-2. COX-1 is the main mediator ensuring gastric mucosal integrity, while COX-2 is mainly involved in inflammation.

As a potent non-steroidal anti-inflammatory drug (NSAID) we have pranoprofen (PRA), chemically known as 2-(5H-chromeno [2,3-b] pyridin-7-yl) propanoic acid, that was authorized for the first time in China by the China food and drug administration (CFDA) in 1999 and is used as an alternative treatment for inflammation in ophthalmic therapy (nonbacterial chronic conjunctivitis, strabismus, cataract surgery, and blepharitis), and as an alternative treatment for osteoarthritis and rheumatoid arthritis [9,10,11,12]. PRA inhibits the cyclooxygenase (COX) enzyme, reducing prostaglandin synthesis from arachidonic acid. Due to its low bioavailability and short plasma half-life, its use is limited and it has an inadequate biopharmaceutical profile [9,13,14].

Skin treatments remain challenging due to the difficulty of controlling the fate of active ingredients (drug substances) in the skin [15,16]. Over the last few decades, nanostructured lipid carriers (NLC) have shown great potential as carriers for the topical administration of active ingredients, mainly due to the possibility of targeted action and controlled release in different skin layers. Many NLCs have poor viscosity and low lipid content, and they need to be thickened to be appropriate for dermal drug administration. It is for this reason that they are incorporated into vehicles, such as creams and gels [17].

Carbomer 940, also known as carbopol^®^ 940, is a cross-linked acrylic acid anionic polymer which increases viscosity, stabilizes emulsions, forms gels and suspends particles in hydrous formulations, is non-toxic, and exhibits minimal or no irritation potential at the concentrations applied in personal care and cosmetics products [18]. It is included in the FDA Inactive Ingredients Guide (oral suspensions and tablets; ophthalmic, rectal, and topical preparations; transdermal preparations; and vaginal suppositories), and it is included in non-parenteral medicines authorized in Europe and in Canada (Canadian List of Acceptable Non-medicinal Ingredients) [19]. Its gelling concentration is 0.5–2.0%. The most common way to achieve maximum thickening of Carbomer polymers is by converting them into a salt, easily accomplished by neutralizing the polymer with sodium hydroxide or triethanolamine. When neutralizing the polymer, the acid groups of the chain become ionized, and they remain negatively charged, which causes the repulsion of the acid groups of the chain, and, consequently, the chain unwinds, and the solution it contains becomes viscous.

Sepigel^®^ 305, also known as Farcosgel, is a product presented as a gelatinous, fluid, yellowish, opalescent dispersion used in gels and gel creams. It consists of polyacrylamide as a gelling agent, polyoxyethylene-7-lauryl ether as a non-ionic emulsifier, and isoparaffin as a fatty oil, which acts as a viscosifier and stabilizer for formulations. It comes in a ready-to-use liquid, pre-neutralized, and is effective over a wide pH range (pH 6.5 at 2%). It is an excellent stabilizer and texturizing agent [20,21]. The concentration as a gelling agent is 2–3% [22].

In previous works, we reported the development of nanostructured lipid carriers loading PRA [23,24]. We further investigated incorporating the PRA-NLC in a thermoreversible hydrogel as a therapeutic agent for local skin inflammatory diseases. The gel resulted in a low-viscosity formulation with limited extensibility and exhibited a sustained release over 80 h [9]. Against this background, this study aimed to develop topical formulations to manage the local inflammation caused by abrasions, focused on obtaining formulations with a fast drug release and improved extensibility profile so the formulation can be applied to the inflamed skin effortlessly for a gentle and painless spreadability. Carbomer 940 and Sepigel^®^ 305 polymers were selected to bear PRA-loaded NLC. Besides the faster drug release and the higher extensibility, the Carbopol-based gel showed a greater amount of PRA retained in the skin after the permeation studies with the predicted plasma concentration at a steady state below the therapeutic range. Thus, it can be assumed that no systemic levels would be achieved and in turn unwanted side effects would be avoided. This study confirms that these formulations developed are a dermal delivery strategy and a safe alternative for modulating PRA release and promoting drug retention in the skin to manage inflammatory skin symptoms locally.

## 2. Results and Discussion

### 2.1. Physicochemical Characterization of the Gels: PRA-NLC-Car and PRA-NLC-Sep 

In order to obtain the desired PRA-NLC suspension, a design-of-experiment (DoE) approach was applied previously using a 2^3^ central composite factorial design as described elsewhere [24]. The effects of the independent variables, such as the concentration of PRA, the concentration of Tween^®^ 80, and the concentration of solid lipids and liquid lipids, on the physicochemical properties were studied. The formulation with the best physicochemical characteristics and highest level of encapsulation efficiency (EE) was selected for further studies. To be concise, the developed PRA-NLC formulation shows a mean particle size of around 248.40 nm and polydispersity index values lower than 0.25, which are characteristic of monomodal systems, and a negative charge with a Zeta potential value of −10.70 mV. The percentage of EE was found to be around 99.7% [9]. These PRA-NLCs were vehiculated in Carbopol and Sepigel formulations, and the physicochemical characterization of such formulations is reported hereafter.

#### 2.1.1. Appearance and pH Evaluation 

Table 1 gives an overview of all formulations prepared fresh at different concentrations of gelling agent (Carbomer 940 or Sepigel^®^ 305). The selected concentrations for our studies were 1% of Carbomer 940 (PRA-NLC-Car) and 3% of Sepigel^®^ 305 (PRA-NLC-Sep) after examining different parameters such as color, homogeneity, consistency, and phase separation.

The PRA-NLC-Car gel and the PRA-NLC-Sep gel exhibited a suitable incorporation of the PRA-NLC, showing white color, smooth consistency, good homogeneous appearance, and no phase separation. These parameters were analyzed after 24 h, 72 h, 7 days, 14 days, and 30 days and no changes were detected (Table 2).

The pH of a dermal formulation is considered an essential factor for its stability and compatibility with the skin, evading dermal irritation and protecting the skin from bacterial infections [25]. The skin’s regular acidity ranges pH from 4 to 6, established on buffering processes in the skin to reduce irritation and depending on the skin area of the body and the age of the person [26]. The pH values were evaluated at 4 °C, 25 °C, and 40 °C and the results indicate that both gels were slightly acidic, asserted in the eudermic range within 5.2 to 6.5 for all values obtained (Table 3), indicating that PRA-NLC-Car and PRA-NLC-Sep are biocompatible with the natural acidity of the skin and suitable for dermal application. Furthermore, they show that the higher temperature (40 °C) did not significantly increase the pH values compared to the lower temperature (4 °C and 25 °C). Moreover, after storing for 30 days at 25 °C ± 2 °C and with a relative humidity of 60% ± 5%, no big changes were detected. These results indicate good stability for both gels.

**Figure 1:**
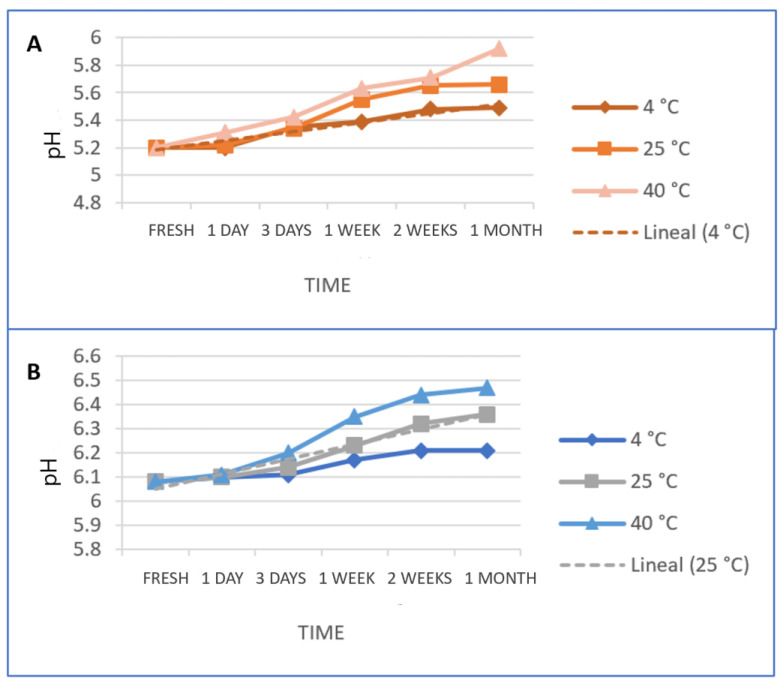
pH values for at 4 °C, 25 °C, and 40 °C for (**A**) PRA-NLC-Car; and (**B**) PRA-NLC-Sep.

Figure 1 shows pH values for PRA-NLC-Car and PRA-NLC-Sep at 4 °C, 25 °C, and 40 °C. Both PRA-NLC-Car and PRA-NLC-Sep show an upward trend in terms of temperature as time goes by. Nevertheless, PRA-NLC-Car at 4 °C and PRA-NLC-Sep at 25 °C seem to fit a linear trend better, meaning these conditions will likely remain stable over time. Nevertheless, it seems to be interesting to detect a time point where temperature suffers significant changes. For this purpose, we used the autocorrelation function (ACF) that measures the correlation of each trial within time.

**Figure 2:**
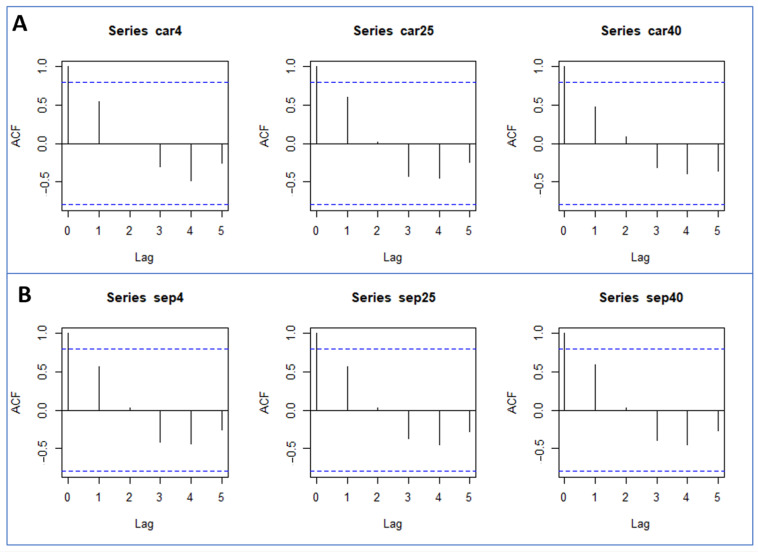
Autocorrelation function plot for the gel formulations at 4 °C, 25 °C, and 40 °C: (**A**) PRA-NLC-Car and (**B**) PRA-NLC-Sep. The blue dotted lines represent a 95 confidence limit of the autocorrelation and partial autocorrelation representations.

Figure 2 shows the autocorrelation function plots for PRA-NLC-Car and PRA-NLC-Sep at 4 °C, 25 °C, and 40 °C. The results of the ACF show that lag 2 has an abrupt change, which means taking a gap of 72 h before/after a new time might start bringing significant changes in the temperature of PRA-NLC-Car and PRA-NLC-Sep.

Series car 4, car 25, and car 40 represent PRA-NLC-Car at 4 °C, 25 °C, and 40 °C, whilst Series sep4, sep25, and sep40 represent PRA-NLC-Sep at 4 °C, 25 °C, and 40 °C. The x-axis represents lagged time points for the given time lapses: fresh, 24 h, 72 h, 7 days, 14 days, and 30 days.

#### 2.1.2. Morphological Characterization

**Figure 3:**
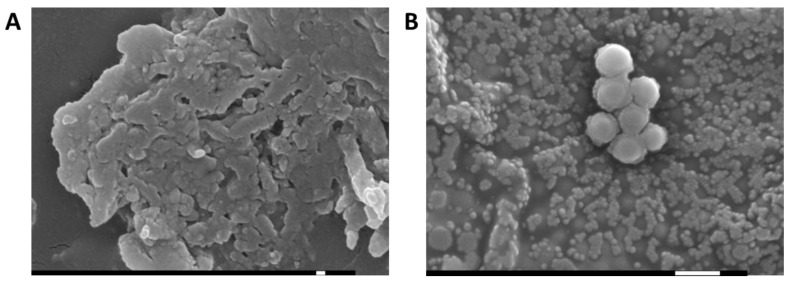
SEM photomicrographs of the dried (**A**) PRA-NLC-Car (magnification ×30,000); and (**B**) PRA-NLC-Sep (magnification ×15,000). Scale bar = 100 nm and 1 µm, respectively.

Figure 3 shows both gels’ morphology and the evaluation of the homogeneity and the possible existence of aggregates observed by scanning electron microscopy (SEM). They had a fiber structure with high porosity, suitable from a drug delivery point of view, which could predict rapid diffusion in the drug delivery tests below.

#### 2.1.3. Fourier-Transform Infrared (FT-IR)

**Figure 4:**
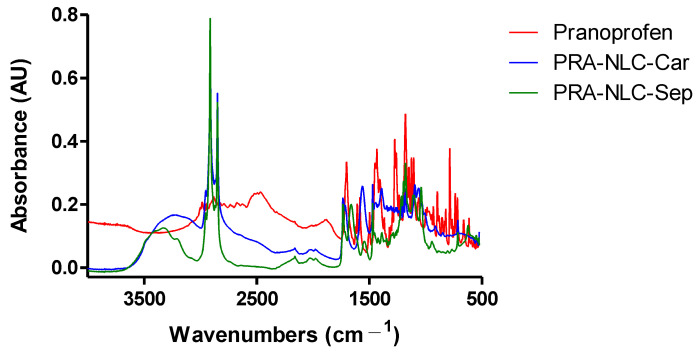
Fourier-transform infrared spectroscopy (FTIR) of PRA-NLC-Car, PRA-NLC-Sep, and PRA.

Fourier-transform infrared spectroscopy (FT-IR) is an effective tool to confirm the analysis of bonds between molecules [27]. Figure 4 shows the spectra of dried PRA-NLC-Car, PRA-NLC-Sep, and PRA. The PRA profile shows the characteristic strong peaks from the C=O bond-stretching vibration at 1700 cm^−1^. C=C stretching of its aromatic rings is presented at 1500 cm^−1^. Other peaks from C=O bonds (between 1650 and 1850 cm^−1^) can also be observed and there are several vibrations observed at the fingerprint region (600–1450 cm^−1^). FT-IR analysis suggests that there is no evidence of new covalent bonds between the drug (PRA) and Precirol^®^ ATO5 and the polymers (Carbomer or Sepigel).

#### 2.1.4. Rheological Profile

**Figure 5:**
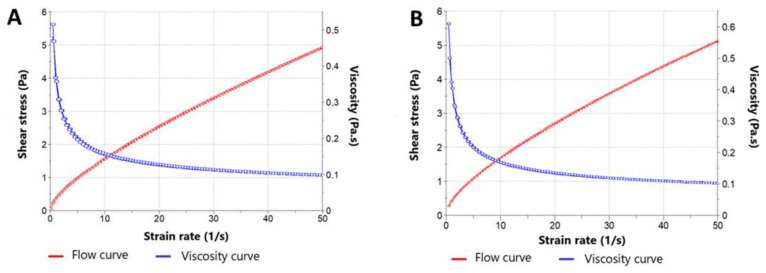
Flow curves and viscosity curves for (**A**) PRA-NLC-Car and (**B**) PRA-NLC-Sep formulations.

For characterizing the flow properties of gels’ forms and forecasting their behavior during manufacturing, packaging, or final use, rheological studies are a valuable tool [28]. The Herschel–Bulkley and Ostwald-de-Waele mathematical models best fit the PRA-NLC-Car data (r^2^ = 0.999), and Ostwald-de-Waele and Cross best fit the PRA-NLC-Sep data (r^2^ = 0.999), which was expected in these types of semisolid formulations and usually clarified the pseudoplastic bearing over a wide range of shear rates. Figure 5 shows the rheological behavior at 25 °C of both formulations. Furthermore, starting from a constant rate cycle of 50 s^−1^, the mean viscosity values obtained 2 days after the production at 25 °C were 9.854 × 10^2^ ± 0.10 mPa·s for PRA-NLC-Car and 10.24 × 10^2^ ± 0.16 mPa·s for PRA-NLC-Sep, proving repeatability between samples (*n* = 3) in the rheological data. 

#### 2.1.5. Spreadability or Extensibility Analysis

**Figure 6:**
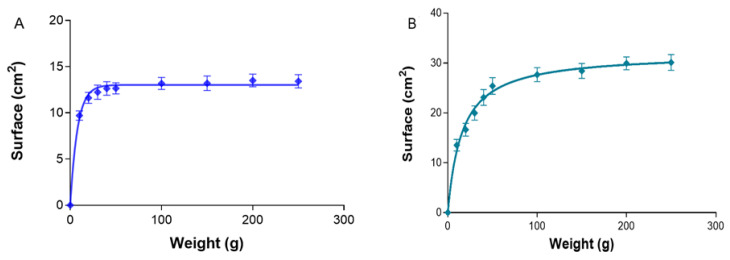
Surface area (cm^2^) on the basis of the tested mass (g) at 25 °C of (**A**) PRA-NLC-Car and (**B**) PRA-NLC-Sep. Mean ± standard deviation (SD) (*n* = 3).

The spreadability property is important because it indicates how the topical forms will behave when extruded from the container (usually a tube) and the application and uniformity of the dose, as a result having an impact on the therapeutic efficacy. Based on the mathematical modeling, PRA-NLC-Car followed a first-order (one-phase exponential association) kinetic profile (Figure 6A), the extensibility values raised proportionally to the loading weight, reaching a value for the maximum extensibility with a maximum release amount (Ymax) = 13.031 ± 0.228 cm^2^, and a release constant (K) of 0.126 ± 0.015 g^−1^. PRA-NLC-Sep followed a hyperbola one-site model (Figure 6B), with a kinetic constant (Kd) of 15.930 ± 1.268 g^−1^ and a value of maximum amount of drug released (Bmax) = 32.010 ± 0.579 cm^2^. The spreadability results of this study indicate that both gels were easily spreadable with the exertion of a small amount of shear [25,29]. 

#### 2.1.6. Swelling, Degradation, and Porosity Studies

**Figure 7:**
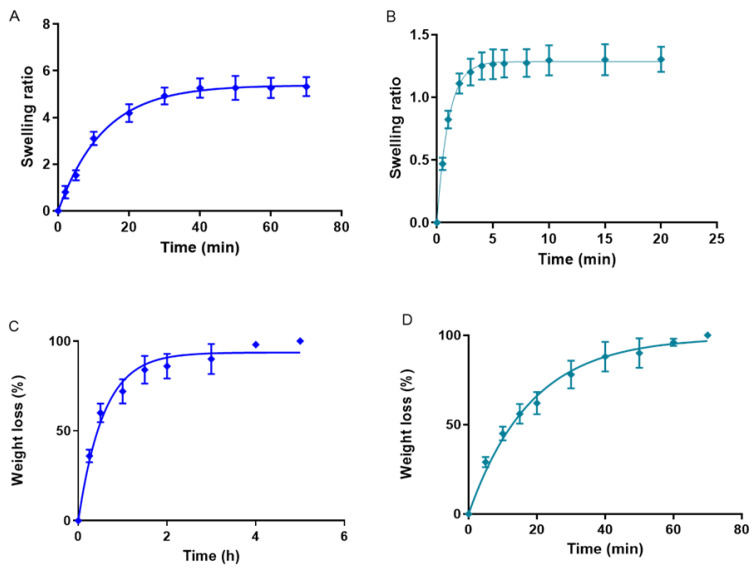
(**A**) The swelling ratio of PRA-NLC-Car; (**B**) swelling ratio of PRA-NLC-Sep, both upon being submerged in PBS; (**C**) representation of the percentage of weight reduction degradation in PRA-NLC-Car; (**D**) percentage of weight loss degradation of PRA-NLC-Sep, both in PBS (pH = 5.5) (*n* = 3).

Swelling studies were performed to assess the capacity of the gels to absorb solvent PBS (pH 5.5) within their structure [30]. Dehydrated gel samples were submerged in PBS (pH 5.5), and their weight was registered at specified intervals. According to the literature, the amount and rate of PBS entrapment are associated with the number of functional groups in the gel’s structure, which affects the fluidity of the polymer chains, thereby reducing or increasing the swelling rate [31]. The swelling processes of PRA-NLC-Car (Figure 7A) and PRA-NLC-Sep (Figure 7B) followed a first kinetic order, described by the kinetic constants with values of K = 0.08 min^−1^ and K = 0.97 min^−1^, respectively. PRA-NLC-Car achieved almost 100% swelling after 40 min and PRA-NLC-Sep after 20 min.

Degradation experiments were carried out by immersing fresh samples of gels in PBS (pH 5.5) and evaluating the weight changes at specified time intervals. Figure 7C,D show the degradation profile of the gels; for PRA-NLC-Car, the total gel was degraded in about 2 h, and for PRA-NLC-Sep in about 50 min, and both followed a first kinetic order. This indicates that the weight reduction in the gels depends on the concentration. Consequently, at more significant concentrations, a greater degradation speed decreases the speed of the degradation process over time. There was a kinetic constant K of 1.75 h^−1^ for PRA-NLC-Car and 0.055 min^−1^ for PRA-NLC-Sep.

The percentage porosity was 84.64% ± 0.17% for PRA-NLC-Car and 76.21% ± 0.16% for PRA-NLC-Sep. It is known that gels with small pore sizes are characterized as having a forceful viscous coupling within the formed network and the solvent, inhibiting the polymer network’s movement relative to the solvent [31,32]. These porosity values are consistent with viscosity values where PRA-NLC-Sep has a lower and high viscosity. In contrast, PRA-NLC-Car is the opposite, with higher porosity associated with lower viscosity.

### 2.2. In Vitro Release Assay

**Figure 8:**
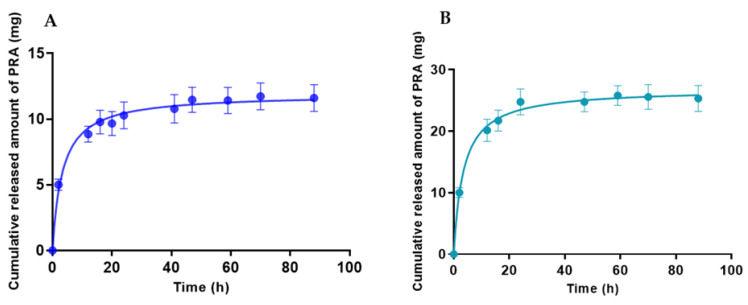
In vitro release profiles of PRA from gels (**A**) PRA-NLC-Car and (**B**) PRA-NLC-Sep.

For both gels, the release profile of PRA follows a nonlinear regression. Figure 8 shows the release profile of PRA. Both formulas follow a hyperbola behavior where PRA-NLC-Sep can release the drug slightly faster than PRA-NLC-Car, with values for Bmax of 26.86 mg and 11.90 mg, respectively. Furthermore, both gels showed similar values of release constant (kd), around 3.4 h (Table 4). The remaining amount of the drug remained trapped in the matrix of the gels.

### 2.3. Ex Vivo Permeation Studies

PRA-NLC-Car and PRA-NLC-Sep were tested ex vivo in human abdominal skin for up to 28 h. Table 5 exhibits the parameters calculated from the permeation, including the theoretical plasma concentration in humans (elderly and young) at the steady state of PRA.

The values of the parameters *J_ss_* (flow) and Kp (permeability coefficient) were calculated from the accumulative permeated amount of PRA (µg) as a function of time. PRA-NLC-Sep showed higher *J_ss_* and Kp. However, the amount of PRA retained in the skin (Q_ret_) is greater with PRA-NLC-Car than PRA-NLC-Sep. Predicted steady-state plasma concentration (*Css*) values obtained for PRA-NLC-Car and PRA-NLC-Sep, taking into account a hypothetical zone of application of 100 cm^2^, were lower than the stated therapeutic plasma concentration corresponding to 4.89 ± 1.29 µg/mL for old-age and 10.19 ± 2.43 µg/mL for young humans [9,31]. As a result of the predicted steady-state plasma concentrations of PRA, both gels will be below therapeutic plasma concentrations. These results ensure that their dermal application has no systemic effects, thus ensuring safe topical anti-inflammatory and analgesic effects.

### 2.4. Anti-Inflammatory Efficacy Studies: Xylol-Induced Mouse Ear Inflammation Model and Rat Back Skin Abrasion Model

#### 2.4.1. Ear Thickness Evaluation and Stratum Corneum Hydration (SCH) 

**Figure 9:**
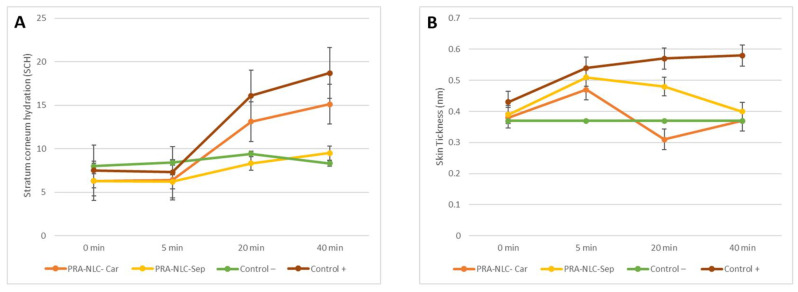
Plots of the biomechanical properties: (**A**) stratum corneum hydration and (**B**) evolution of the skin thickness of the mice ears over time.

A mouse ear inflammation model induced by topical application of xylol was used to assess the anti-inflammatory activity of PRA-NLC-Car and PRA-NLC-Sep. Table 6 shows the skin thickness of the mouse ears, and Table 7 shows stratum corneum hydration values up to 40 min. Figure 9 shows the stratum corneum hydration results (SCH) and the differences in the ear thickness, symbolizing the inflammatory reaction and the inflammatory decrease.

Table 8 shows the results of the cross-correlation function of PRA-NLC-Car, PRA-NLC-Sep, and control + between the skin thickness and the stratum corneum hydration. This concludes that there is no strong relationship between the measures of skin thickness and stratum corneum hydration at any point in the experiment. The behavior of these two experiments develops independently.

#### 2.4.2. Histological Studies

**Figure 10:**
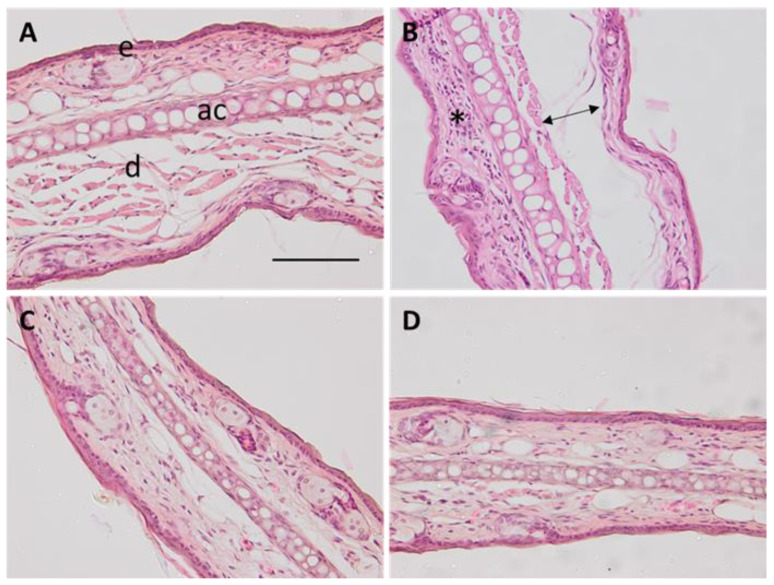
Representative images of histological sections of mice ears treated with different formulations: (**A**) negative control; (**B**) positive control, (**C**) PRA-NLC-Car; and (**D**) PRA-NLC-Sep. The asterisk indicates leucocyte infiltrate, the arrow indicates disruption due to edema, e is the epidermis, d is the dermis, and ac is the auricular cartilage, 200× magnification. Scale bar = 100 µm.

Mice’s ears were exposed to xylol to induce inflammation of the tissue. As can be observed in Figure 10B, a mild infiltration of leucocytes and a disruption of the tissue due to edema appeared after the exposition. However, animals treated with PRA-NLC-Car or PRA-NLC-Sep topically (Figure 10C,D, respectively) did not show those signs due to the prevention of inflammation. This indicates that both formulations containing PRA decrease the level of leucocyte infiltration.

**Figure 11:**
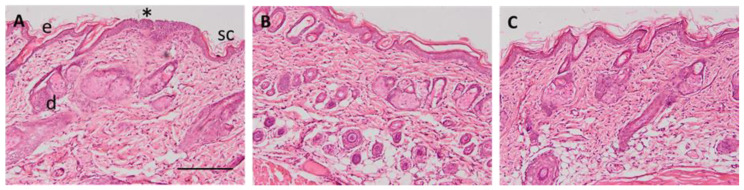
Representative images of histological sections of rat skin treated with different formulations: (**A**) negative control; (**B**) PRA-NLC-Car; and (**C**) PRA-NLC-Sep. Asterisk indicates a scar-increased epidermis and loss of stratum corneum. SC is the stratum corneum, e is the epidermis, and d is the dermis. Images at 100× magnification. Scale bar = 200 µm.

Additionally, hairless rat back skins were exposed to microneedle abrasion, and the skin histology in control conditions shows the scar after the procedure (Figure 11A). In Figure 11B,C, the back skin was exposed to PRA-NLC-Car or PRA-NLC-Sep, and no signs of inflammation could be observed following the treatment. Both formulations prevented the loss of stratum corneum and decreased the infiltration of inflammatory cells.

### 2.5. In Vivo Tolerance and Biomechanical Human Skin Properties Evaluation

**Figure 12:**
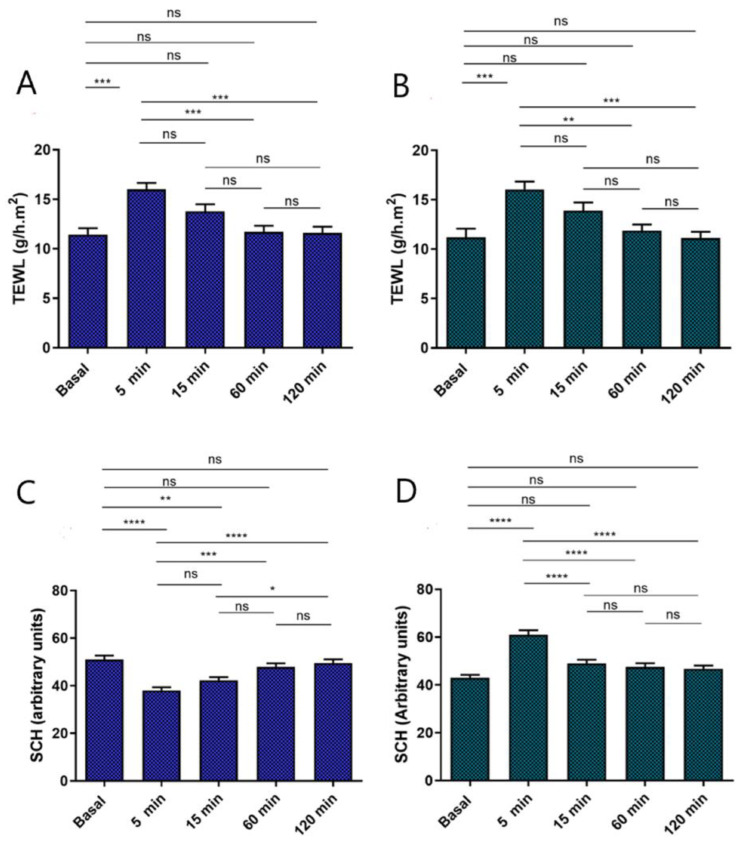
Biomechanical parameters evolution was monitored before applying the gels, and 5 min, 15 min, 1 h, and 2 h after application. (**A**) TEWL of PRA-NLC-Car (g/h × m^2^); (**B**) PRA-NLC-Sep (g/h × m^2^); (**C**) SCH of PRA-NLC-Car (arbitrary units); (**D**) SCH of PRA-NLC-Sep (arbitrary units). Significant statistical differences: **** *p* < 0.0001, *** *p* < 0.001, ** *p* < 0.01, * *p* < 0.05, ns = non-significant.

Figure 12 illustrates the development of the monitored biomechanical parameters pre and post applying the gels for up to 120 min. This study is to assess the effect of the selected gels on skin hydration and their integrity. The Transepidermal Water Loss (TEWL) values obtained, that grew significantly at 5 min post application, decreased at 30 min post application, and remained invariable (Figure 12A,B). These results indicate that both gels are not occlusive topical formulations. The stratum corneum hydration (SCH) results acquired from PRA-NLC-Car showed a decrease 5 min post application and they gradually raised until stable (Figure 12C), and SCH values obtained from PRA-NLC-Sep presented an increase 5 min after the application and then remained unchanged for up to 2 h (Figure 12D). Considering that skin capacitance is directly corelated to skin hydration, these values suggest that PRA-NLC-Sep barely increases the hydration in relation to the regular behavior of the human skin. No visible signals of skin irritation were detected post dermal application of both gels on volunteers, demonstrating that the selected gels were well tolerated dermally.

## 3. Conclusions

The present work demonstrates that the two gels developed (PRA-NLC-Car and PRA-NLC-Sep) were well characterized physiochemically, with good stability at different temperatures and well tolerated for dermal application. Moreover, the skin barrier function was preserved, and no visible irritation was identified. Additionally, PRA- NLC-Sep could release the drug faster than PRA-NLC-Car, which showed higher values for *J_ss_* and Kp. However, the PRA-NLC-Car showed higher values of Q_ret_ on the skin. Taking into consideration the in vivo study, both formulations showed excellent anti-inflammatory activity, with a reduction in leukocytic infiltrate after the topical treatment. These results suggest that either one or the other formulation could be used, depending on the inflammatory skin disease, with excellent results. This article brings to light two new topical formulations containing encapsulated PRA, which could be used for a variety of skin inflammatory processes such as abrasion caused by depilation.

## 4. Materials and Methods

### 4.1. Reagents

Pranoprofen (CAS 52549-17-4) was acquired from Alcon Cusi (Barcelona, Spain). Castor oil (CAS 8001-79-4) (*Ricinus communis* L.) and Tween^®^ 80 (CAS 9005-65-6) (Polyoxyethylene 20 sorbitan monooleate) were obtained from Sigma-Aldrich Química (Barcelona, Spain). Precirol^®^ ATO 5 (CAS 8067-32-1) (Glyceryl Palmitostearate) and LAS (CAS 61791-29-5) (PEG-8 Caprylic/Capric Glycerides) were obtained from Gattefosse (Saint-Priest, France). Carbomer 940 (CAS 9003-01-4) (acrylic acid polymer) was purchased from Fagron ibérica (Barcelona, Spain). Sepigel 305^®^ (CAS 64742-47-8) (Polyacrylamide, C13-14 Isoparaffin Laureth-7) was purchased from Acofarma (Barcelona, Spain). The purified water utilized in all experiments was collected from a MilliQ^®^ Plus System (Darmstadt, Germany), lab-supplied. The tablets of phosphate-buffered saline (PBS) were acquired from Sigma-Aldrich Chemie (Steinheim, Germany) as well as processed as specified by the manufacturer and stored in fridge for further use. All other chemicals for analytical experiments and reagents utilized in the research were of analytical or high-performance liquid chromatography (HPLC) grade and acquired from Fisher Scientific (Leicestershire, UK).

### 4.2. Preparation of PRA-NLC Dispersion

PRA-NLC was prepared by a high-pressure homogenization technique [32,33], optimized, and described previously [24]. The hot lipid phase (5 wt % regarding the total amount of formulation), containing Precirol^®^ ATO 5 as solid lipid, LAS/castor oil (75/25) as liquid lipids, and 1.5 wt% of PRA, was melted in a water bath at 85 °C to obtain a clear homogeneous lipid phase solution. This lipid phase was added to an aqueous phase solution containing Tween^®^ 80 that was heated simultaneously at the same temperature. A dispersion (premix) was formed using an ultra turrax (T25 IKA Ultra-Turrax, Staufen, Germany) for 45 s at 8000 rpm. The dispersion was passed thrice through a high-pressure homogenizer (12800 FPG, Stansted, UK) at 85 °C and 800 bar. The obtained nanoemulsion was cooled down to 25 °C (room temperature) to recrystallize the lipid and form the PRA-NLC. The PRA-NLC formulation was optimized by a factorial design 2^3^, described previously [24]. Previous studies reported all the characterization regarding hydrodynamic mean diameter, polydispersity index, zeta potential, and encapsulation efficiency [23,24].

### 4.3. Preparation of the Gels: PRA-NLC-Car and PRA-NLC-Sep Gels

- PRA-NLC-Car: The blank gel was developed with carbomer 940 (1% *w*/*v*), dispersed in milli Q water, and permitted to hydrate for a day. Afterward, it was stirred moderately and then the pH was adjusted with triethanolamine to ensure a eudermic pH. The PRA-NLC suspension previously optimized and selected was incorporated into the blank gel under continuous magnetic stirring (final formula composition 10.7 mg PRA/g gel). It was left without stirring until it reached equilibrium for a day at 25 °C (room temperature) prior to use.
- PRA-NLC-Sep: The blank gel was prepared with Sepigel 305^®^ (3% *w*/*v*) dispersed in milli Q water under continuous stirring to obtain the gel formulation. The PRA-NLC was added to the gel under magnetic stirring until complete homogenization with a final formula composition of 10.7 mg PRA/g gel to maintain an effective active ingredient concentration. The gel was left without stirring right up until achieved equilibrium for a day at 25 °C (room temperature) prior to use. Table 9 indicates the percentage composition for the different phases during of the formulations, as well as the total percentage in the final product.

### 4.4. Physicochemical Characterization of PRA-NLC-Car and PRA-NLC-Sep

#### 4.4.1. pH and Morphological Characterization

Values of pH of PRA-NLC-Car and PRA-NLC-Sep gels were measured by a calibrated digital pH-metre micro (pH 2001 Crison Instruments SA, Alella, Spain). It is useful to check gels’ compatibility and the adequacy for use on damaged skin and as a mechanism to support the stability of the gels [34].

Small samples of PRA-NLC-Car and PRA-NLC-Sep were dried over 9 days in a vacuum desiccator to examine their morphology. When completely dried, a small quantity was coated using a slim carbon layer as a conductive agent [20] with an Emitech K-950 coater (Quorum Tech. Kent, UK). By scanning electron microscopy (SEM), the interior structure of the developed gels was visualized using a JEOL 7100FE-JSM (Peabody, MA, USA).

#### 4.4.2. Fourier-Transform Infrared (FT-IR)

To obtain FT-IR spectra of dried PRA-NLC-Car, PRA-NLC-Sep, and PRA, a Thermo Scientific Nicolet iZ10 with an ATR diamond and DTGS detector were used. The scanning range was 525–4000 cm^−1^.

#### 4.4.3. Rheological Behavior

Forty-eight hours after preparation of the gels, rheological measurements were determined in triplicate with a rotational rheometer (Haake Rheostress 1, Thermo Fischer Scientific, Karlsruhe, Germany) provided with a cone–plate setup (the gap between cone and plate was 0.106 mm), with a plate and a movable higher cone Haake C60/2° Ti (60 mm diameter, 2° angle). The rheometer was equipped with a Thermo Haake Phoenix II + Haake C25P temperature controller device (Thermo Fischer Scientific, Waltham, MA, USA) and operated by a HaakeRheowin^®^ Job Manager v. 4.0 (Thermo Fischer Scientific, Waltham, MA, USA) for testing and HaakeRheowin^®^ Data Manager v.4.0 (Thermo Fischer Scientific, Waltham, MA, USA) for analysis of the acquired data. Viscosity and flow curves were measured at 25 °C. The conditions of the shear rate ramp program adjusted a 3 min ramp-up period from 0 to 50 s^−1^, a 1 min uniform-shear-rate period from 50 s^−1^, and a ramp down from 50 to 0 s^−1^ for 3 min. The rotational flow curve values were fitted to various mathematical model equations: Newton, Ostwald-de-Waele, Bingham, Casson, Cross, and Herschel–Bulkley [20]. The best fit of mathematical models was based on the correlation coefficient value (r). The mean viscosity value (Pa·s) was evaluated from the uniform share section at 50 s^−1^ for each formulation.

#### 4.4.4. Spreadability or Extensibility Analysis

**Figure 13:**
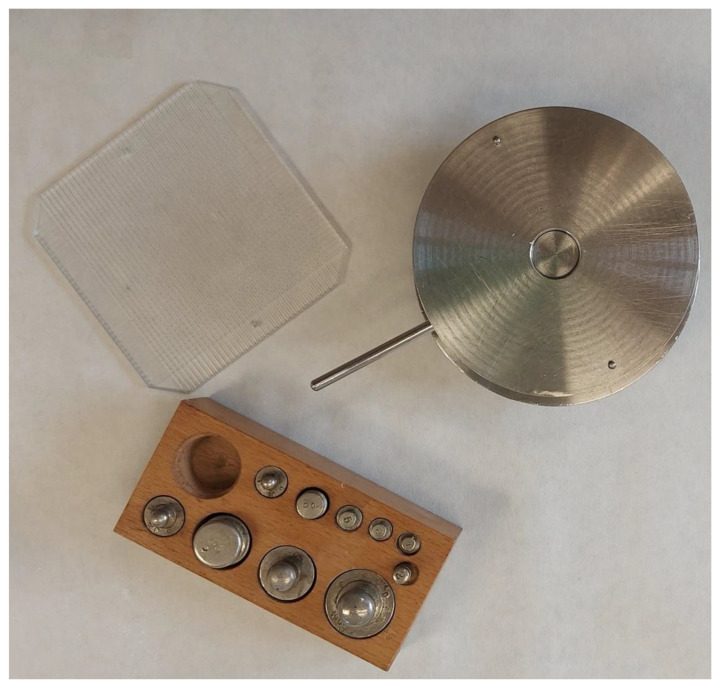
Extensibility device.

Extensibility was determined by placing samples of 0.5 g of each gel (PRA-NLC-Car and PRA-NLC-Sep) between a two-platform device (Figure 13) (the glass platform located in the upper position is pre-marked) and observing the area over which the samples extended, forcing them to spread after applying on the top platform several incremental, specific, standard weights (10, 20, 30, 40, 50, 100, 150, 200, and finally 250 g) and leaving them to stand on top of the glass platform for 90 s. We measured the expansion in diameter and recorded it as a function of the specific weight applied. The results were revealed as spread area versus applied mass according to the following equation [35,36]:

(1) $$\;\;\;\;S=d^{2}\times \frac{\pi }{4}\;$$

where *S* is the spreading area or extensibility (cm^2^) computed from the utilized mass of sample (g), and d is the average diameter (cm) impacted by the sample. Experimental results were analyzed according to the best kinetic model and fitted to different mathematical models with the statistical program GraphPad Prism^®^ v.8.0.0, GraphPad Software, San Diego, CA, USA. The r value confirmed the model fitting.

#### 4.4.5. Swelling, Degradation, and Porosity Studies

The parameters of swelling ratio (*SR*) and the degradation experiment (which was expressed as the percentage of weight loss (WL)) were evaluated based on a gravimetric method. Samples of dried gel were used to carry out the swelling test, and samples of fresh gel were used for the degradation test. In both trials, the samples were immersed in PBS (pH = 5.5) at a temperature of 32 °C for 30 min and were withdrawn and weighed, subsequently blotting the surface water at predetermined intervals. The *SR* ratio was calculated based on Equation (2) and represented by a kinetic model:

(2) $$SR=\frac{Ws-Wd}{Wd}\;$$

where *Ws* denotes the mass (weight) of the swollen PRA-NLC-Car and PRA-NLC-Sep samples at specified times, and *Wd* is the mass (weight) of dried gels.

The degradation rate of the gel was determined as the percentage of weight loss (*WL*) with the following equation and represented by a kinetic model:

(3) $$WL\;\left(\%\right)=\frac{Wi-Wd}{Wi}\times 100\;$$

where *Wi* is the initial mass (weight) of the samples of PRA-NLC-Car and PRA-NLC-Sep after each immersion after specified interval times and *Wd* is the mass (weight) of the gel at various time points.

The porosity percentage was assayed based on the sinking of the PRA-NLC-Car and PRA-NLC-Sep samples after drying in absolute ethanol for 20 min and weighing them every 2 min when the excess was blotted. The porosity (*P*) of the gels was calculated using the following expression:

(4) $$\;\;\;\;\;\;\;\;\;\;\;P\;\left(\%\right)=\frac{W2-W1}{ð\times V}\times 100$$

where *W*2 denotes the mass (weight) of the samples after each immersion in ethanol every 2 min, *W*1 is the mass (weight) of the dried samples, ð = density of the ethanol, and *V* = volume of the sample used.

### 4.5. In Vitro Release Assay

In vitro release assays were conducted with amber vertical glass Franz diffusion cells [37,38] (400 FD; Crown Glass, Somerville, NJ, USA) equipped with dialysis membranes (Dialysis Tubing Visking, Medicell Intl. Ltd., London, UK). Firstly, the membranes were hydrated in a solution of methanol/water (6:4; *v*/*v*) for a day, after previously being mounted in the Franz cell between the donor and receptor compartment. The receptor used was phosphate-buffered saline (PBS) (pH 7.4) kept with continuous stirring at 700 rpm for assuring sink conditions. A summary of the experimental conditions is given in Table 10. Samples of PRA-NLC-Car PRA-NLC-Sep were placed in the donor compartment. Samples of 200 µL were collected with a syringe from the receptor compartment at predefined times and the volume withdrawn was replaced by an equal volume of PBS (pH 7.4). The aliquots were analyzed by RP-HPLC as reported in previous studies [23,24].

The amount of PRA released was adjusted to different kinetic models; the best model-fitting aptness was selected by calculating the value of the coefficient of determination (r^2^) [39].

### 4.6. Ex Vivo Permeation Study

Ex vivo human skin permeation was performed using the same Franz diffusion cells as in in vitro release studies [37,38]. The skin was stored frozen at −20 °C and was dermatomed (Aesculap, GA630, Tuttlingen, Germany) at 0.4 mm thick pieces and fixed, separating the donor and receptor chambers [40].

The skin diffusion area was 0.64 cm^2^, and PBS (pH 7.4) was utilized to fill the receptor chamber. The samples of the gels were added to the donor chamber directly in contact with the skin and covered with parafilm^®^ to prevent evaporation. An amount of 200 µL of samples was withdrawn from the receptor chamber at predetermined time points and replaced by an equal amount of the receptor medium. Table 11 shows the experimental conditions used for the ex vivo permeation study.

Once the experiment was complete, the skin was removed from the cells and rinsed with 0.05% sodium lauryl sulfate and purified water to eliminate the gel on the skin’s surface. The permeation area of the skin in direct contact with the formulation was cut out, weighed, and immersed in methanol/water (1:1, *v*/*v*) and sonicated in an ultrasound bath for 25 min to extract the total retained quantity of PRA from the pieces of skin (Q_ret_). The quantity of PRA retained and permeated was determined by HPLC [31].

The steady-state flux across the skin (*J_ss_*, µg/h/cm^2^) and the transdermal permeability coefficient (*K_p_*, cm/s) were calculated according to the following equations [41]:

(5) $$J_{ss}=\frac{Qt}{A}\cdot t\;$$

(6) $$K_{p}=\frac{J_{ss}}{C_{0}}$$

in which *Qt* (μg) is the quantity (amount) of drug permeated across the skin and thus detected in the receptor chamber, *A* (cm^2^) denotes the active cross-sectional area (accessible for diffusion), *t* (h) is the time of exposure, and *C*_0_ is the primary drug concentration in the donor chamber.

In view of the pharmacokinetic parameters of the PRA for young and old-age humans, the estimated steady-state plasma theoretical concentration *C_ss_* of the drug that would penetrate the skin post topical administration was estimated with the following equation:

(7) $$\;C_{ss}=J_{ss}\;\cdot \;\frac{A}{CLp}$$

where *J_ss_* is the flux, *A* is the hypothetical area of application (considering in this case 100 cm^2^), and *CL_p_* is the human plasma clearance of PRA (reported values of 609.00 cm^3^/h and 1146.60 cm^3^/h, for old-age and young humans, respectively) [31,42].

### 4.7. Anti-Inflammatory Efficacy Studies: Xylol-Induced Mouse Ear Inflammation Model and Rat Back Skin Abrasion Model

#### 4.7.1. Study Protocol

The in vivo studies were performed based on the Academic Ethics Committee of the Nursery of the Autonomous University of the State of Morelos (Mexico) that approved the Study Protocol on 19 January 2023, and the Official Mexican Standard for the Care and Management of Animals (code: NOM-062-ZOO-1999).

Male *Mus musculus* mice (10–12 weeks; 12–30 g) (*n* = 9) were used for the mouse ear inflammation model. The animals were permanently maintained under a controlled temperature and photoperiod (light between 06:00 and 18:00 h). Food and water were available ad libitum.

The inflammatory process was induced topically by applying xylol to the right ear for 40 min [41]. Three inflamed mice were chosen as a group for the + control. A second group received the PRA-NLC-Car gel treatment after xylol application (*n* = 3), and a third group received the PRA-NLC-Sep treatment (*n* = 3). In addition, a fourth group of untreated, healthy mice was used as a negative control (*n* = 3). The treatment was applied twice for one day. Finally, mice were sacrificed by cervical dislocation, and the right ears were excised for histological studies. Female hairless rats (7–9 weeks; 70–75 g) (*n* = 9) were used in the rat skin model for the abrasion test. The animals were permanently maintained under a controlled temperature and photoperiod (light between 6:00 a.m. and 6:00 p.m.). Food and water were available ad libitum.

The damage was induced topically on the back and by applying abrasion with a special glove. Three damaged rats were used as a group for control (*n* = 3). A second group was damaged and given treatment with PRA-NLC-Car gel (*n* = 3), and the other group was damaged and provided treatment with PRA-NLC-Sep (*n* = 3). The treatment was applicated twice for one day. Finally, rats were sacrificed and the backs were cut for histological studies.

#### 4.7.2. Ear Thickness Evaluation and Stratum Corneum Hydration (SCH)

Skin thickness and SCH parameters of the mice ears were measured before inflammation, after inflammation with xylol, and after treatment with PRA-NLC-Car or PRA-NLC-Sep. For measurement of the SCH, a CM-825 Corneometer (Courage & Khazaka Electronics GmbH, Köln, Germany) was used, and for measurement of the thickness a digital thickness gauge of 0 to 10 mm (Mitutoyo Corp, Kawasaki, Japan) was used.

#### 4.7.3. Histological Studies

After applying the formulations, mice and rats were sacrificed. The mice’s ears were obtained, and the skin of the rats’ backs was surgically excised to study the anti-inflammatory effect of the formulations. The tissues were soaked in 4% buffered formaldehyde for 24 h, dehydrated in a gradient of ethanol, and finally embedded in melted paraffin. Blocks were cut into 5 mm pieces, stained with hematoxylin and eosin, and viewed under a microscope (Olympus BX41 and Olympus XC50 camera).

### 4.8. In Vivo Tolerance, Biomechanical Human Skin Properties Evaluation

#### 4.8.1. Transepidermal Water Loss (TEWL)

In vivo skin human tolerance studies by evaluating biomechanical properties were authorized by the University of Barcelona Ethics Committee on 30 January 2019 (IRB00003099).

Total skin water loss (TEWL) was evaluated with a Tewameter^®^ (TM 300 Courage-Khazaka Electronics GmbH, Cologne, Germany), which measures the amount of water entering the environment (atmosphere) around the epidermal layer of the skin through diffusion and evaporation processes [29,43]. Ten healthy-skinned participants were recruited and were asked not to use skin-care cosmetics on the flexor side of the left forearm for 24 h before the measures. Small circles were drawn, and records were compiled (baseline records), and then a homogeneous layer of approximately 0.5 g of the gels PRA-NLC-Car and PRA-NLC-Sep was applied to the center of the circle. New measurements were collected after application and at 5, 15 min, 1 h, and 2 h post application. To measure, the probe was pressed and held on the selected area of skin for 60 s. TEWL data (g/m^2^·h) were expressed as mean ± SD (*n* = 10).

#### 4.8.2. Hydration Measurements (SCH)

The evaluation of the hydration of the stratum corneum (SCH) was carried out with an 825 Corneometer^®^ (Courage & Khazaka Electronics GmbH, Cologne, Germany) and was determined before application in the basal state and 5, 15 min, 1 h and 2 h post application of PRA-NLC-Car and PRA-NLC-Sep gels on the treated area. The measurements were carried out using the capacitance method, which takes advantage of water’s relatively high dielectric constant compared to other skin substances. SCH data (arbitrary units) are expressed as the mean ± SD (*n* = 10).

## Figures and Tables

**Table 1:** Physical evaluation of concentrations of the formulations with different concentrations of gelling agent for the parameters color changes, homogeneity, consistency, and phase separation.

	Concentration (%)	Color	Homogeneity	Consistency	Phase Separation
	0.5	White	++	++	None
Carbomer 940 gels	1	White	+++	+++	None
	2	White	+++	++	None
	2	White	++	++	None
Sepigel^®^ 305 gels	2.5	White	++	+++	None
	3	White	+++	+++	None

+++ (excellent); ++ (good); (+) fair.

**Table 2:** Evaluation of the stability of PRA-NLC-Car and PRA-NLC-Sep.

	Stability Parameters	Fresh	1 Day	3 Days	1 Week	2 Weeks	1 Month
	Color	White	White	White	White	White	White
PRA-NLC-Car	Odor	−ch	−ch	−ch	−ch	−ch	−ch
	Phase separation	−ch	−ch	−ch	−ch	−ch	−ch
	Homogeneity	−ch	−ch	−ch	−ch	−ch	−ch
	Color	White	White	White	White	White	White
PRA-NLC-Sep	Odor	−ch	−ch	−ch	−ch	−ch	−ch
	Phase separation	−ch	−ch	−ch	−ch	−ch	−ch
	Homogeneity	−ch	−ch	−ch	−ch	−ch	−ch

−ch (no change); +ch (change).

**Table 3:** pH values for PRA-NLC-Car and PRA-NLC-Sep at 4 °C, 25 °C, and 40 °C.

	Temperature	Fresh	1 Day	3 Days	1 Week	2 Weeks	1 Month
	4 °C	5.20	5.20	5.35	5.39	5.48	5.49
PRA-NLC-Car	25 °C	5.20	5.22	5.34	5.55	5.65	5.66
	40 °C	5.20	5.31	5.42	5.63	5.71	5.92
	4 °C	6.08	6.10	6.11	6.17	6.21	6.21
PRA-NLC-Sep	25 °C	6.08	6.10	6.14	6.23	6.32	6.36
	40 °C	6.08	6.11	6.20	6.35	6.44	6.47

**Table 4:** Values for the parameters fitting and goodness of fit assessed in accordance with one-site hyperbola equation for release profiles of PRA-NLC-Car and PRA-NLC-Sep. Bmax is the maximum extensibility predicted by the hyperbola model and Kd is the time at which the extensibility equals half of the maximim.

One-Site Binding (Hyperbola) (Y = Bmax × X/Kd + X)	PRA-NLC-Car	PRA-NLC-Sep
Best-fit values		
Bmax (mg)	11.90	26.86
Kd (h)	3.352	3.476
Std. Error		
Bmax	0.2619	0.5332
Kd	0.5259	0.4629
95% CI (profile likelihood)		
Bmax	11.37 to 12.43	25.77 to 27.96
Kd	2.280 to 4.425	2.522 to 4.429
Goodness of Fit		
Degrees of Freedom	31	25
R squared	0.9500	0.9706
Square Sum	20.17	58.04
Sy.x	0.8066	1.524

**Table 5:** Drug biopharmaceutical permeation parameters upon ex vivo study and calculation of the predicted steady-state plasma concentration (*C_ss_*) of the PRA after considering applying PRA-NLC-Car and PRA-NLC-Sep on 100 cm^2^ of skin. Results are stated as the median and (minimum–maximum) range values (*n* = 5).

	*J_ss_* (µg/h/cm^2^)	K_p_ (cm/h) × 10^3^	Q_24h_ (µg)	Q_ret_ (µg/g/cm^2^)	Old-Age Humans *C_ss_* (µg/mL) × 10^4^	Young Humans *C_ss_* (µg/mL) × 10^4^
PRA-NLC-Car	0.02917	1.9447	0.88	134.15	47.901	25.442
	(0.02745–0.03163)	(1.8302–2.1087)	(0.82–0.93)	(130.09–138.32)	(45.074–51.938)	(23.940–27.585)
PRA-NLC-Sep	0.31781	21.1875	12.66	79.98	5.218	2.771
	(0.30275–0.33054)	(20.1833–22.0361)	(12.07–12.99)	(75.23–91.43)	(4.971–5.428)	(2.640–2.883)

**Table 6:** Skin thickness (nm) of the mouse ears.

	0 min	5 min	20 min	40 min
PRA-NLC-Car	0.38 ± 0.01	0.47 ± 0.02	0.31 ± 0.01	0.37 ± 0.01
PRA-NLC-Sep	0.39 ± 0.03	0.51 ± 0.02	0.48 ± 0.02	0.40 ± 0.03
Control −	0.37 ± 0.02	0.37 ± 0.01	0.37 ± 0.01	0.37 ± 0.01
Control +	0.43 ± 0.04	0.54 ± 0.05	0.57 ± 0.06	0.58 ± 0.05

**Table 7:** SCH (stratum corneum hydration) (arbitrary units) of the mouse ears.

	0 min	5 min	20 min	40 min
PRA-NLC-Car	6.3 ± 0.01	6.4 ± 0.01	13.1 ± 0.02	15.1 ± 0.02
PRA-NLC-Sep	6.3 ± 0.03	6.2 ± 0.04	8.3 ± 0.04	9.5 ± 0.04
Control −	8 ± 0.01	8.4 ± 0.02	9.4 ± 0.02	8.3 ± 0.02
Control +	7.5 ± 0.03	7.3 ± 0.02	16.1 ± 0.04	18.7 ± 0.3

**Table 8:** Results of the cross-correlation function of PRA-NLC-Car, PRA-NLC-Sep, and control + between the skin thickness and the stratum corneum hydration.

	0	1	2	3
PRA-NLC-Car	−0.66	−0.113	0.369	0.054
PRA-NLC-Sep	−0.282	−0.574	0.06	0.201
Control +	0.744	−0.056	−0.372	−0.202

**Table 9:** Percent composition of PRA-NLC, blank gels, and the PRA-NLC-loaded gels.

Phase	Ingredient	Percentage (%)	Total Percentage in Final Formula (%)	
			PRA-NLC-Car	PRA-NLC-Sep
NLC	Pranoprofen	1.50	1.07	1.07
	Tween^®^80	2.50	1.79	1.79
	Solid lipid (Precirol ATO5)	2.50	0.45	0.45
	Liquid lipid (LAS) ^1^	1.88	1.34	1.34
	Liquid lipid (Castor oil)	0.63	1.79	1.79
	Milli-Q water	91.00	64.99	64.99
Car-Gel	Carbomer 940	1.00	0.29	-
	Triethanolamine	0.10	0.03	-
	Milli-Q water	98.90	28.27	-
Sep-Gel	Sepigel 305	3.00	-	0.86
	Milli-Q water	97.00	-	27.72

^1^ LAS: PEG-8 Caprylic/Capric Glycerides.

**Table 10:** Resume of experimental conditions for the in vitro release study.

Parameter	Condition
Receptor fluid	Phosphate-buffered saline (PBS pH = 7.4)
Cell volume	5 mL
Membrane	Dialysis membrane
Diffusion area	0.64 cm^2^
Temperature	32 ± 0.5 °C
Stirring	600 r.p.m.
Dose	2.6 g
Sample volume	200 µL
Sampling times	0, 12, 16, 20, 24, 41, 47, 59, 70, 88 h
Replicates	*n* = 5

**Table 11:** Resume of the experimental conditions for the ex vivo skin permeation test.

Parameter	Condition
Receptor medium	Phosphate-buffered saline (PBS pH = 7.4)
Franz cell volume	5 mL
Membrane	Abdominal human skin
Skin diffusion area	0.64 cm^2^
Thickness	400 µm
Temperature	32 ± 0.5 °C
Stirring	600 r.p.m.
Dose	0.1 g
Sample volume	200 µL
Sampling times	4, 10, 18, 22, 25, 28, and 34 h
Replicates	*n* = 5

## Data Availability

The data presented in this study are available on request from the corresponding author.
